# Short-Term Neurodevelopmental Outcome in Term Neonates Treated with Phenobarbital versus Levetiracetam: A Single-Center Experience

**DOI:** 10.1155/2019/3683548

**Published:** 2019-06-02

**Authors:** Raffaele Falsaperla, Laura Mauceri, Piero Pavone, Massimo Barbagallo, Giovanna Vitaliti, Martino Ruggieri, Francesco Pisani, Giovanni Corsello

**Affiliations:** ^1^Neonatal Intensive Care Unit, Santo Bambino Hospital, University Hospital “Policlinico-Vittorio Emanuele”, Via Tindaro 2, 95124 Catania, Italy; ^2^Unit of Pediatrics and Pediatric Emergency, University Hospital “Policlinico-Vittorio Emanuele”, Via Plebiscito 628, 95124 Catania, Italy; ^3^Department of Pediatrics, AOU Policlinico-Vittorio Emanuele, University of Catania, Via S. Sofia 78, 95123 Catania, Italy; ^4^Department of Pediatrics, Azienda Ospedaliera di Rilievo Nazionale e di Alta Specializzazione Garibaldi, Catania, Italy; ^5^Unit of Rare Diseases of the Nervous System in Childhood, Department of Clinical and Experimental Medicine, Section of Pediatrics and Child Neuropsychiatry, University of Catania, 95124 Catania, Italy; ^6^Child Neuropsychiatry Unit, Medicine & Surgery Department, Neuroscience Division, University of Parma, Parma, Italy; ^7^Department of Maternal and Child Health, University of Palermo, Palermo, Italy

## Abstract

**Background:**

Phenobarbital (PB) has been traditionally used as the first-line treatment for neonatal seizures. More recently, levetiracetam (LEV) has been increasingly used as a promising newer antiepileptic medication for treatment of seizures in neonates.

**Objectives:**

The aim of our study was to compare the effect of PB vs. LEV on short-term neurodevelopmental outcome in infants treated for neonatal seizures.

**Method:**

This randomized, one-blind prospective study was conducted on term neonates admitted to the Neonatal Intensive Care Unit of S. Bambino Hospital, University Hospital “Policlinico-Vittorio Emanuele,” Catania, Italy, from February 2016 to February 2018. Thirty term neonates with seizures were randomized to receive PB or LEV; the Hammersmith Neonatal Neurological Examination (HNNE) was used at baseline (T0) and again one month after the initial treatment (T1).

**Results:**

We found a significantly positive HNNE score for the developmental outcomes, specifically tone and posture, in neonates treated with LEV. There was no significant improvement in the HNNE score at T1 in the neonates treated with PB.

**Conclusion:**

This study suggests a positive effect of levetiracetam on tone and posture in term newborns treated for neonatal seizures. If future randomized-controlled studies also show better efficacy of LEV in the treatment of neonatal seizures, LEV might potentially be considered as the first-line anticonvulsant in this age group.

## 1. Introduction

Seizures are one of the most frequent neurological disorders during the first 28 days of life with an incidence of 2.29 per 1000 live births [1].

The detrimental effect of seizures on the neurodevelopmental outcome of newborns is well recognised, further highlighting the need for a safe antiepileptic medication without negative effect on the infants' development [2].

The most common antiepileptic drugs used in Neonatal Intensive Care Units (NICU) are phenobarbital (PB) and phenytoin (PHT) [3, 4]. According to the Guidelines on Neonatal Seizures, published in 2011, the first choice of treatment for neonatal seizures remains PB [5–7], even though PB is effective in less than 50% of cases [8–10]. Moreover, PB is associated with several side effects, among them, its negative effect on psychomotor development and neurological outcomes [11, 12]. For these reasons, therapeutical alternatives for neonatal seizures have been explored. Among the new antiepileptic drugs (AEDs) [13, 14], levetiracetam (LEV) has been approved as add-on therapy for the treatment of focal seizures in patients over 4 years of age in Europe. LEV appears to have an excellent tolerability in neonates [15, 16]. Its antiepileptic effect is based on the binding of the synaptic vesicle protein 2 (SV2) at the presynaptic terminal [17]. LEV appears to have good efficacy; a favourable safety profile and its rapid intravenous administration are not associated with cardiovascular adverse effects [18].

One of the uncommon side effects described in therapy with LEV was increased irritability and tiredness [19, 20] whereas therapy with PB can induce sedation and respiratory depression.

In animal models, LEV also seems to be safer than PB. PB exposure with dosages similar to those used in humans seems to induce neuronal apoptosis in the developing rat brain [21], whereas LEV does not induce cell death [22]. PB, but not LEV, has been found to interfere with maturation of synaptic connections [23, 24]. Increased schizophrenic-like behavioural outcome was reported in rats treated with PB [25]. No neurotoxic effect of levetiracetam was found in the developing rat brain [22], and a neuroprotective effect on hypoxic-ischemic brain injury in neonatal rats or rodent was confirmed by several studies [17, 22, 26–28].

Clinical studies have reported concerns of significant cognitive and motor impairments related to PB exposure, especially in pediatric populations [12, 29]. Potential neurotoxicity of PB has also been raised.

To date, there are only few reports of using LEV for the treatment of neonatal seizures, and its effects on developmental outcomes in particular remain unknown. To our knowledge, only the study by Maitre et al. looked at the developmental outcomes of the antiepileptic treatment as the primary objective. This study showed negative effects of PB but a positive association between the use of LEV and improved neurodevelopmental outcomes [30].

Here, we report the results of our randomized, one-blind prospective study conducted on two groups of term neonates with seizures, who were treated with either LEV or PB. The neurodevelopmental outcomes were measured using the Hammersmith Neonatal Neurological Examination (HNNE), at baseline and again after 1 month of treatment.

## 2. Materials and Methods

### 2.1. Study Design

This was a randomized, one-blind prospective study conducted on term newborns receiving PB or LEV as first-line treatment for seizure control. We included patients admitted to the Neonatal Intensive Care Unit (NICU), S. Bambino Hospital, University Hospital “Policlinico-Vittorio Emanuele,” Catania, Italy, between February 2016 and February 2018. In this period, 4636 deliveries were recorded in our Hospital.

We included term neonates with seizures manifesting within the first 28 days of life. The seizure semiologies included clonic or tonic-clonic seizures, ocular abnormal movements, and subtle motor manifestations, such as tongue thrusting, cycling limb movements, or apnea.

We classified neonatal seizures into 3 main categories according to the ILAE criteria on neonatal seizures: (a) “acute symptomatic seizures (ASS),” caused by acute diseases, including asphyxia, stroke, meningoencephalitis, and other acute lesions, such as those secondary to the hypoxic ischemic disease; (b) seizures secondary to chronic structural brain abnormalities (congenital malformations, encephalomalacia, and other cerebral lesions), defined as “structural epilepsy” (SE); and (c) seizures secondary to a genetic condition (ionic channel diseases, vitamin-dependent epilepsies, or other epileptogenic diseases without underlying structural abnormalities), defined as “genetic epilepsy” (GE) [31].

Newborns with SE, GE, and seizures secondary to transient metabolic disorders, including hypoglycemia and hypocalcemia; neonates with a positive history for maternal drug ingestion; those who received more than one anticonvulsant medication; and those neonates in whom LEV was used as second-line therapy were excluded to make the clinical sample as homogeneous as possible.

The underlying etiologies for seizure onset included hypoxic-ischemic encephalopathy not requiring therapeutical hypothermia, stroke, and central nervous system (CNS) infections.

In all the patients, the onset of seizures was in the first 72 hours of life. 50% of the patients experienced reduction in the seizure burden (SB) within the first 6 hours from AED initiation, and 100% were seizure-free 1 week after introducing the treatment. Therapy was maintained for one month after the seizures resolved.

All neonates underwent a clinical and diagnostic evaluation at baseline (T0), before starting the anticonvulsant treatment, and then again after 1 month of therapy (T1). The short-term neurological outcomes were measured using the Hammersmith Neonatal Neurological Examination.

In order to evaluate the safety of PB vs. LEV, we recorded the onset of any emerging adverse event, including neurological symptoms and kidney and renal function alterations. None of the patients needed mechanical respiratory assistance.

At baseline, the following data were collected: patients' demographic data, familial and maternal gestational history, age of neonates, and clinical description of signs and/or symptoms, with inclusion and exclusion criteria evaluation.

A complete laboratory assessment including glucose, electrolytes, urine toxicology screen, thyroid hormones, and metabolic screening (serum amino acid levels, blood ammonia, and urinary level of fatty acids) was performed. Head ultrasounds were performed on all neonates before and after the treatment. All included patients underwent serial video-EEG recordings.

The participants became eligible for the study after their parents signed a study consent form, by which they agreed to the diagnostic and therapeutic intervention, as well as to the study data collection.

The study protocol conformed to the ethical guidelines of the 1975 Declaration of Helsinki as revised in 2000 [32] and was approved by the ethic committee of the University of Catania, Italy.

All authors declare not to present any conflict of interest in the publication of the present study.

### 2.2. Treatment Protocols

Newborns were randomly assigned to receive PB and LEV in a blinded manner. The drugs were administered at the following doses: intravenous (IV) PB with an initial dose of 20 mg/kg, followed by a maintenance dose of oral PB at 5 mg/kg; IV LEV at an initial dose of 20 mg/kg, followed by a maintenance dose of oral LEV at 20 mg/kg, with gradually increasing doses up to 40 mg/kg twice daily in case of nonresponse at initial doses. PB was administered according to the Italian Society of Neonatology guidelines [33]. LEV was administered according to the recommendations published by Yau et al. in 2015 [34].

### 2.3. Outcome Measures

We performed the Hammersmith Neonatal Neurological Examination (HNNE) at baseline (T0) and after one month of treatment (T1). The Hammersmith Neonatal Neurological Examination (HNNE) developed by Dubowitz and Dubowitz is widely used in newborns for their neurological assessment [35]. The Hammersmith score was evaluated by trained neonatologists of our NICU, who evaluated the following neurological items: (1) tone and posture, (2) tone patterns, (3) movements, (4) reflexes, (5) abnormal signs, and (6) orientation and behaviour.

### 2.4. Statistical Analysis

For statistical evaluation, we used dedicated software: JMP (product of SAS Institute Inc., Cary, NC 27513-2414, USA) and GraphPad 5.0 (La Jolla, CA, USA). We reported qualitative variables as percentage and quantitative ones as mean ± standard deviation. For those variables presented as mean ± standard deviation, normal distribution was checked by the Kolmogorov-Smirnov one-sample test and statistics for kurtosis and symmetry. The chi-square test was used to compare qualitative variables. The Student *t*-test was used to compare quantitative results.

*P* values under 0.05 were considered statistically significant.

## 3. Results

We included 30 neonates, 12 females and 18 males, with a mean gestational age of 38.30 ± 1.30 weeks. The demographic data of the two groups are shown in Table 1. All patients were affected by acute symptomatic seizures (ASS) and particularly stroke, CNS infection, and hypoxic-ischemic encephalopathy not requiring therapeutic hypothermia.

The neurological assessment, the seizures types, and the Hammersmith score performed at baseline in the two groups are shown in Table 2. The Hammersmith score and each single item showed better scores at T1, with significant differences between T0 and T1 (*P* = 0.001) in the LEV group. Among the single items, tone and posture, reflexes, orientation, and behaviour showed a statistical improvement. No significant differences between T0 and T1 were reported in the PB group (Table 3).

**Figure 1:**
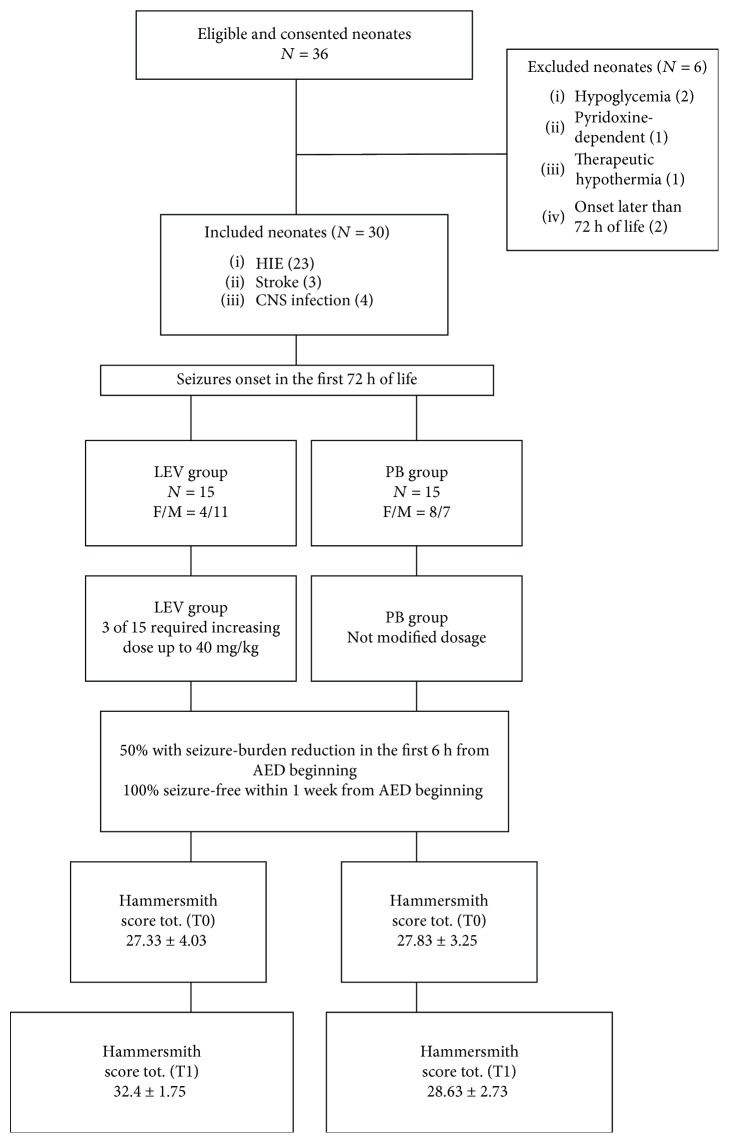
Flow diagram of recruitment, outcomes, and antiepileptic drugs (AED) used in two cohorts.

In Figure 1, we summarize in a flow chart the clinical features of our patients and the achieved results.

## 4. Discussion

Our study found that neonates with seizures who were treated with LEV showed better HNNE test scores 1 month after treatment initiation, compared to neonates treated with PB.

The HNNE is a test developed for the clinical assessment of term and preterm infants at risk of developmental delay. It is a specific and predictive test that evaluates posture and tone, reflexes, movements, and neurobehavioral responses. Literature data showed that the HNNE test is able to predict cerebral palsy with a sensitivity range of 57-86% and specific range of 45-83% when carried out before term age [35].

In 2018, a first systematic review on the efficacy of LEV in the treatment neonatal seizures was published by McHugh et al. [36]. Their study was the first to examine the efficacy of LEV compared to PB in neonates. The authors demonstrate the clinical equipoise between LEV and PB in the setting of neonatal seizures. They conclude that LEV does not appear to be neurotoxic and it may potentially offer fewer and/or less severe long-term cognitive effects, when compared to phenobarbital with its known, potentially neurotoxic effects.

Two large multicentric studies of intravenous LEV use are currently under way. The first, LEVNEONAT, is a multicenter French clinical trial with the aim to develop new treatment strategies for the treatment of neonatal seizures using levetiracetam. The purpose of this study is to determine the correct dosing, safety, and efficacy of the intravenous levetiracetam as a first-line treatment in term newborns with seizures secondary to HIE. Their first clinical data seem to confirm that LEV is a promising treatment for seizures in newborns [37, 38]. The aim of the second study is to determine the efficacy of intravenous LEV, as a first-line anticonvulsant for treatment of neonatal seizures, compared to phenobarbital. Seizure burden will be based on duration and frequency of seizure events [39].

To date, there are only few clinical studies focused on neonatal seizures treated with LEV.

Hmaimess et al. [40] reported on a neonate with seizures unresponsive to traditional therapy. Abend et al. [16] described 24 neonates with seizures where the treatment with LEV at maintenance doses of 10-80 mg/kg/day resulted in clinical improvement. Khan et al. [19] treated neonates with seizures using IV LEV at the dose of 25 mg/kg/day. Ramantani et al. [20] extended their study to 38 newborns with seizures. Rakshasbhuvankar et al. [18] treated eight neonates with IV LEV.

Falsaperla et al. [41] reported on 16 neonates, 12 born at term and 4 preterm. Neonates responded to treatment with a variable range of seizure resolution ranging from 24 h to 15 days. Twelve neonates with seizures were studied by Yau et al. [34]. LEV has also been administered as adjunctive therapy by Shoemaker and Rotenberg [42] in three neonates with seizures treated with PB and PHT, PHT, and PHT plus CMZ. In all patients, LEV proved to be effective without adverse effects. In Table 4, we summarize the results of these reports in more detail.

To our knowledge, only the study by Maitre et al., a single large retrospective study of 280 infants, whose seizures were treated with either LEV or PB, compared the effects of these drugs on the neurodevelopment outcome [30]. The authors included all patients with at least one witnessed clinical seizure who received PB or LEV. They assessed neurodevelopmental outcomes by measuring motor, cognitive, and language performance on the Developmental Assessment of Young Children (DAYC) at 12 months of age and by using the Bayley Scales of Infant Development (BSID) at 24 months. Their study suggested that exposure to PB might be associated with worse neurodevelopmental outcomes at 2 years of age and that LEV may be associated with improved outcomes compared to PB. Regarding cognitive and motor scores the effect were less evident with LEV.

However, none of the reported studies explored the short-term influence of the antiepileptic drugs measured by the neurological examination of the newborns one month after the initiation of the treatment. In our study, we demonstrated an improvement of the short-term neurological outcomes in neonates treated with LEV compared to those treated with PB. Our results suggest that LEV, with its presumed neuroprotective action and safer side-effect profile, might represent a good alternative for the treatment of neonatal seizure especially in those patients with an abnormal neurological examination.

Nevertheless, further clinical studies are needed to prove the efficacy of LEV in neonatal age, given its benefits on the neurological development of these patients.

### 4.1. Limitation

Our study has a few limitations. First is the small number of patients. We also included patients with a variety of etiologies within the group of acute symptomatic seizures (ASS). Lastly, a limitation of the study is also the lack of a long-term outcome.

However, we believe that despite the limitations, our study might serve as a first step in the development of larger double-blind placebo-controlled trials in order to assess for potentially protective effect of LEV.

## Figures and Tables

**Table 1:** Demographic data.

	LEV group	PB group	*P* value, Student *t*-test, and chi-square test
Number of patients	15	15	
Gestational age	38.13 ± 1.24	38.33 ± 1.04	NS
Sex (F/M)	4/11	8/7	
Prenatal anomalies	40%	40%	NS
APGAR score 1 min.	7.66 ± 1.29	8.66 ± 0.89	NS
APGAR score 5 min.	9.13 ± 1.12	9.03 ± 0.84	NS
Respiratory distress	33.33%	40%	NS

**Table 2:** Neurological assessment, seizures types, EEG study, and Hammersmith score of the LEV and PB groups before treatment.

Orientation and behaviour	5.45 ± 1.03	5.33 ± 0.97	0.41
	LEV group	PB group	*P* value
Neurologic examination	Abnormal: 46%	Abnormal: 40%	0.31

Seizures types	Automatism: 13.33%	Automatism: 6.66%	
	Tonic seizures: 26.66%	Tonic seizures: 20%	
	Multifocal clonic seizures: 20%	Multifocal clonic seizures: 26.66%	
	Focal clonic seizures: 40%	Focal clonic seizures: 40%	
		Autonomic seizures: 6.66%	

EEG background study			
Discontinuity	85%	90%	
Burst suppression	15%	10%	

Hammersmith score			
Hammersmith score (tot.)	27.33 ± 4.03	27.83 ± 3.25	0.21
Tone and posture score	7.5 ± 1.18	7.6 ± 0.96	0.40
Tone pattern score	4.3 ± 0.64	4.33 ± 0.72	0.57
Movements	2.56 ± 0.49	2.73 ± 0.45	0.58
Reflexes	4.86 ± 0.89	4.76 ± 0.59	0.06
Abnormal signs	2.8 ± 0.41	2.93 ± 0.25	0.90
Orientation and behaviour	5.16 ± 1.01	5.45 ± 1.03	0.42

**Table 3:** Changes in the Hammersmith score in the PB group and in the LEV group.

	PB group before treatment	PB group after treatment	*P* value
Hammersmith score (tot.)	27.83 ± 3.25	28.63 ± 2.73	0.26
Tone and posture score	7.6 ± 0.96	8.03 ± 0.93	0.45
Tone pattern score	4.33 ± 0.72	4.30 ± 0.61	0.27
Movements	2.73 ± 0.45	2.86 ± 0.35	0.17
Reflexes	4.76 ± 0.59	4.9 ± 0.47	0.20
Abnormal signs	2.93 ± 0.25	2.98 ± 0.30	0.74
Orientation and behaviour	5.45 ± 1.03	5.33 ± 0.97	0.41

	LEV group before treatment	LEV group after treatment	*P* value
Hammersmith score (tot.)	27.33 ± 4.03	32.4 ± 1.75	**0.001**
Tone and posture score	7.5 ± 1.18	9.36 ± 0.76	**0.05**
Tone pattern score	4.3 ± 0.64	4.75 ± 0.46	0.11
Movements	2.56 ± 0.49	3.1 ± 0.5	0.52
Reflexes	4.86 ± 0.89	5.56 ± 0.49	**0.01**
Abnormal signs	2.8 ± 0.41	3.1 ± 0.5	0.76
Orientation and behaviour	5.16 ± 1.01	6.7 ± 0.45	**0.02**

**Table 4:** 

Source	Study type	Population	Gender distribution	Primary LEV	Secondary LEV	Loading dose	Maintenance dose	Other AED
Hmaimess et al. (2006)	Case report	Term neonate	1M	0	1	10 mg/kg	30 mg/kg	PHT, CLZ, PB, MDZ, LMT
Shoemaker and Rotenberg (2007)	Case report	Preterm/term	1F/2M	0	3	60 mg/kg	30 mg/kg	PB, PHT, MDZ
Abend et al. (2011)	Retrospective	Preterm/term neonates	11M/12F	4	19	10-20 mg/kg	5-40 mg/kg	PB, PHT, TPM, MDZ, FA
Ramantani et al. (2011)	Prospective	Preterm/term neonates	24M/14F	38	0	10 mg/kg	Up to 60 mg/kg	Up to 2 doses of PB 20 mg/kg
Khan et al. (2011) [43]	Retrospective	Term neonates	10M/12F	3	19	10-50 mg/kg	25 mg/kg	PB, FP, LZP, MDZ
Khan et al. (2013)	Retrospective	Preterm neonates	4M/7F	3	8	25-50 mg/kg	25 mg/kg	PB
Rakshasbhuvankar et al. (2013)	Case series	Preterm/term neonates	5M/3F	0	8	5-10 mg/kg	10-35 mg/Kg	PB, CZP, TPM, OXC
Yau et al. (2015)	Retrospective	Preterm/term neonates		0	12	7-20 mg/kg	5-60 mg/kg	PHT, TPM
Falsaperla et al. (2017)	Prospective	Preterm/term neonates		16	0	10 mg/kg	Up to 40 mg/kg	PB

Legend: CLZ: clonazepam; FA: folinic acid; FP: fosphenytoin; LZP: lorazepam; MDZ: midazolam; OXC: oxcarbazepine; PB: phenobarbital; PHT: phenytoin; TPM: topiramate.

## Data Availability

The data used to support the findings of this study may be released upon application to the corresponding author who can be contacted at raffaelefalsaperla@hotmail.com.
